# Empirical Evidence Reveals Seasonally Dependent Reduction in Nitrification in Coastal Sediments Subjected to Near Future Ocean Acidification

**DOI:** 10.1371/journal.pone.0108153

**Published:** 2014-10-16

**Authors:** Ulrike Braeckman, Carl Van Colen, Katja Guilini, Dirk Van Gansbeke, Karline Soetaert, Magda Vincx, Jan Vanaverbeke

**Affiliations:** 1 Ghent University, Department of Biology, Marine Biology Research Group, Ghent, Belgium; 2 Netherlands Institute for Sea Research, Department of Ecosystem Studies, Yerseke, The Netherlands; Auckland University of Technology, New Zealand

## Abstract

Research so far has provided little evidence that benthic biogeochemical cycling is affected by ocean acidification under realistic climate change scenarios. We measured nutrient exchange and sediment community oxygen consumption (SCOC) rates to estimate nitrification in natural coastal permeable and fine sandy sediments under pre-phytoplankton bloom and bloom conditions. Ocean acidification, as mimicked in the laboratory by a realistic pH decrease of 0.3, significantly reduced SCOC on average by 60% and benthic nitrification rates on average by 94% in both sediment types in February (pre-bloom period), but not in April (bloom period). No changes in macrofauna functional community (density, structural and functional diversity) were observed between ambient and acidified conditions, suggesting that changes in benthic biogeochemical cycling were predominantly mediated by changes in the activity of the microbial community during the short-term incubations (14 days), rather than by changes in engineering effects of bioturbating and bio-irrigating macrofauna. As benthic nitrification makes up the gross of ocean nitrification, a slowdown of this nitrogen cycling pathway in both permeable and fine sediments in winter, could therefore have global impacts on coupled nitrification-denitrification and hence eventually on pelagic nutrient availability.

## Introduction

Over the past 250 years, the atmospheric CO_2_ concentrations have increased by nearly 40% as a consequence of human activities [1]. This increase is partly counteracted by the capacity of the oceans to absorb CO_2_, which occurs currently at a rate of about 10^6^ metric tons of CO_2_ per hour [2]. The latter process led to a decrease in surface-ocean pH by about 0.1 units since the start of the industrial revolution [3]. Climate change models predict a further decrease of 0.35 units by the end of the century for open ocean waters [3], [4] and recent measurements for coastal zones even reveal acidification rates that are an order of magnitude higher [5], [6].

This decrease in pH is known to have a direct or indirect negative effect on many nektonic, pelagic and benthic organisms [7]–[10] and thus marine food web structures in general [11]. Although knowledge on the effects of ocean acidification on the level of organisms and populations is increasing fast, the understanding of how biogeochemical processes are affected in acidified seawater is lagging behind [12]. As the ocean carbon cycle is linked with the cycles of major nutrient elements, it is to be expected that ocean acidification will have large consequences for marine ecosystem functioning [13], [14]. With respect to the N-cycle, currently available knowledge suggests that ocean acidification will lead to increased N_2_ fixation while nitrification may decrease [13]. This would reduce the supply of oxidized nitrogen substrate to denitrifiers and reduce levels of nitrate-supported primary production that may instigate shifts in plankton communities [13]. Most empirical evidence illustrates that ocean acidification decreases nitrification rates in pelagic environments [15]–[18] but the consequences for N-cycling in soft-sediments underlying an acidified water column are far less understood. As coastal sediments are spatially and temporally heterogeneous, the available studies do not allow a proper generalisation, e.g. due to the absence of seasonal replication and the limited amount of sediment types investigated [19]–[25]. Part of the benthic variability in coastal soft-sediments is linked to the macrobenthic communities that vary both spatially (e.g. sediment type) and temporally (e.g. seasonal demographics). Furthermore, the impact of these communities and species on biogeochemical cycling might fluctuate seasonally [26]–[28] and depends on the environmental context such as temperature or algal bloom deposition. A decrease in seawater pH has been shown to affect both macrobenthic bioturbation and bio-irrigation activities [18], [22], [24], which alter redox gradients and microbial communities that regulate the cycling of energy and matter.

To investigate ocean acidification effects on benthic nitrogen cycling and organic matter mineralization, we measured multiple response variables associated with benthic nitrogen cycling in closed core incubations, using two different sediment types in control and manipulated sea water carbonate chemistry. These incubations were executed during two seasons of the year representing the period prior to the annual phytoplankton bloom and the actual bloom period. Nitrification consumes NH_x_ (NH_4_^+^+NH_3_) and O_2_, and produces NO_x_ (NO_2_^−^+NO_3_^−^) thus any effect of acidification on nitrification may be reflected in the fluxes of these substances, as well as alter the oxygen penetration depth in the sediment. We constructed integrated mass budgets of O_2_, NO_x_ and NH_x_ [29] to estimate nitrification rates from the measured fluxes.

## Material and Methods

### Sampling and experimental set-up

We sampled a fine sandy sediment station (St. 115bis: 51° 09.2′N; 02° 37.2′E, 13 m depth) and a station with coarse, permeable sediment (St. 330: 51° 26.0′N; 02° 48.5′E, 20 m depth), before (February) and during (April) the annual phytoplankton bloom of 2012. Both stations are located in the subtidal part of the Belgian Part of the North Sea, characterized by turbid waters with a light extinction coefficient of 0.36 m^−1^ [30], excluding light penetration to the sea floor, hence also precluding microphytobenthos growth. Sampling was carried out with the *RV* Simon Stevin. At each station, a CTD cast was performed to record the water temperature.

Sediment was collected by means of a Reineck box-core at both stations. At St. 115bis with fine sandy sediment (median grain size 180 µm with 14% of mud; [31]), four separate Reineck box-corers were subsampled with a single Plexiglass tube each (internal diameter ø: 10 cm; H: 25 cm). At St. 330 with permeable sediment (median grain size 360 µm, devoid of mud; permeability: 5.3 10^−10^ m^2^, [31]), we measured fluxes in centrally stirred chambers (Plexiglass, ø: 19 cm; H: 30 cm) to create a pore water flow similar to *in situ* conditions [32]. As these chambers were too large to insert in the Reineck box-core, they were filled with homogenized sediment from the upper 10 cm of the sediment from 7 Reineck box-corers. The experimental collections differed between the two sites rendering them not strictly comparable. No specific permits were required for the described field study: the location is not privately-owned or protected in any way and the field study did not involve endangered or protected species.

**Figure 1:**
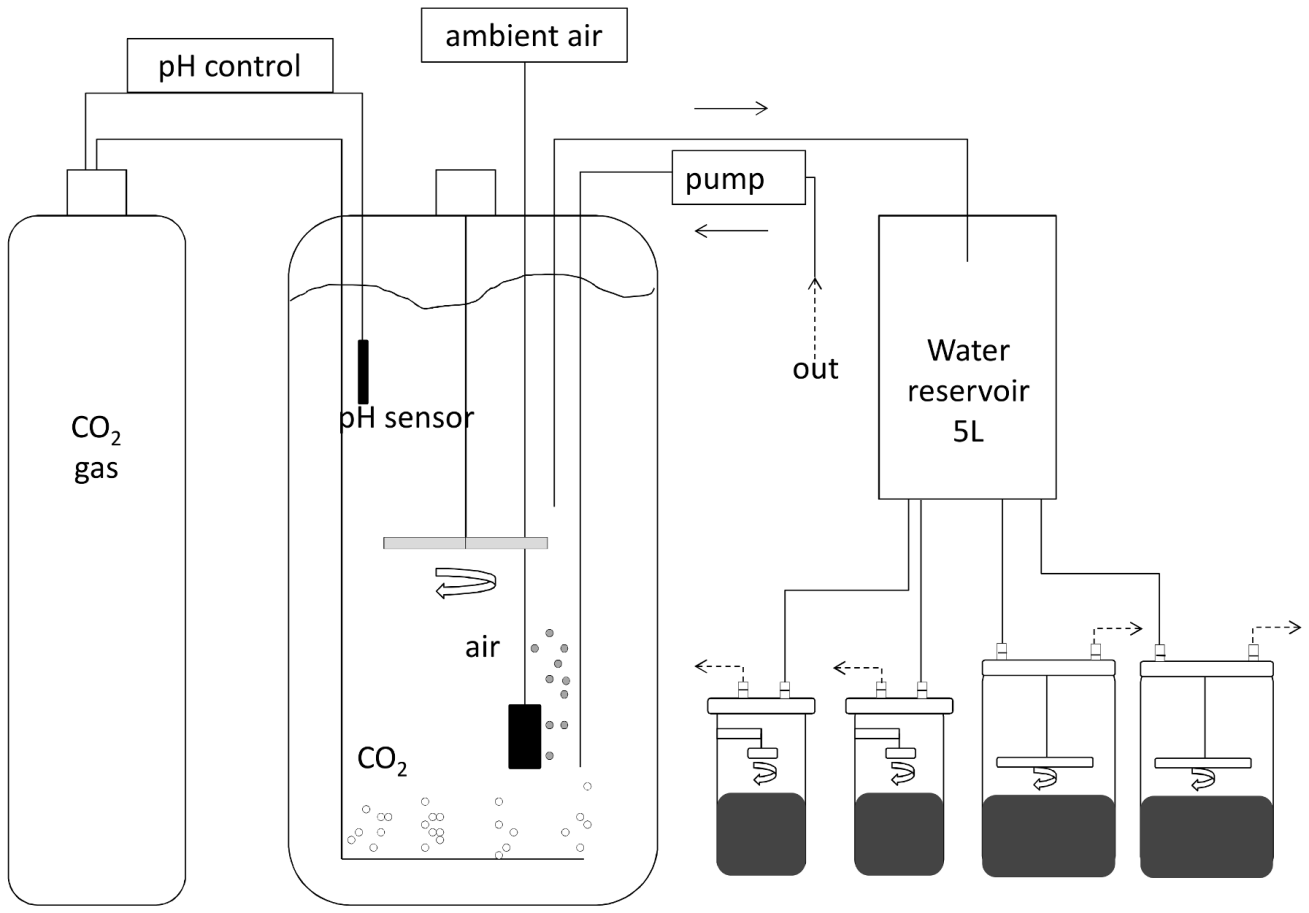
Scheme of experimental set-up. Dashed arrows on core outflow represent tubes that lead to the pump, directing them back into the tank. See also text for explanation. The same set-up without the CO_2_-gas was used as a control.

Sediment cores were transported within 10 h to a temperature-controlled room at *in situ* temperature (recorded from the CTD cast: February: 5°C, April: 9°C) in the lab and immediately submerged in well-aerated sea water in two set-ups (one control set-up and one set-up to be acidified; Fig. 1). Teflon coated magnets rotated by a central magnet were adjusted 5 cm above the sediment surface of the fine sandy sediment cores to ensure water mixing. The rate of water circulation was kept well below the resuspension threshold. Lids equipped with a flat stirring disc were fixed onto the permeable sediment chambers with the stirring disc rotating at 12 rpm 5.4 cm above the sediment surface. From 200 L tanks, a flow-through of seawater through inlet and outlet ports in the lids was ensured with a peristaltic pump (Watson-Marlow 520S), refreshing the core volume ca. 2× h^−1^. The outflow of the cores was recycled and aerated in the 200 L tanks. Our set-up encompassed duplicate cores for each pH treatment – sediment type combination in each month.

### Manipulation of carbonate chemistry

After 24 h of acclimatization, the acidification process was started. To create a predicted pH likely to establish within the current century in coastal waters (∼pH 7.7; [33]), the carbonate chemistry of natural sea water was manipulated through controlled pumping of 100% CO_2_ gas at the bottom of a 200 L seawater tank (∼pH 8.0), using Dulcometer technology (ProMinent) connected to a pH electrode (Dulcotest PHE-112SE) mounted in the tank [33] (Fig. 1). This method was chosen as it best replicates ocean acidification by altering dissolved inorganic carbon (DIC) while keeping total alkalinity constant [34]. Every other day the pH electrode was calibrated using Hanna Instruments' pH NBS buffers. The control seawater unit had the same set-up except for the CO_2_ gas supply and was bubbled with ambient air.

The sediments were incubated under experimental conditions for 14 days in the dark. During this acclimatization period, temperature, salinity (WTW COND 330), pH (Hanna Instruments), oxygen (Pyroscience needle-type optodes) and alkalinity (Gran titration [35], were monitored daily. Carbonate system parameters (*p*CO_2_, dissolved inorganic carbon and calcite and aragonite saturation states) of the overlying water in each tank for the experiment duration were calculated from alkalinity, pH, temperature and salinity in CO2SYS [36] and can be found in Table S1.

### Sediment core incubations

We quantified sediment community oxygen consumption (SCOC) and dissolved inorganic nitrogen (DIN) exchange using a direct flux approach by incubating undisturbed sediment cores. From the resulting oxygen and DIN fluxes, we modeled nitrification rates using an integrated mass budget approach [28], [29].

One week after acclimatization, depth profiles of oxygen were measured in the sediment with O_2_ microsensors (100 µm tip size, Unisense) in vertical increments of 250 µm (3 replicates in each core), up to 10 mm depth. pH profiles were measured in depth intervals of 1000 µm with pH microsensors (tip size 500 µm, Unisense), starting just above the sediment surface downwards, until a constant pH was reached (max. 3.5 cm). A two-point calibration was performed in pH buffers 4.01 and 7.00 before the measurements. Electrodes were connected to a picoammeter (oxygen) or pH-meter and output was displayed on an online PC using SensorTracePro (Unisense) software.

After a 14-day acclimatization period, dark incubations were initiated. Sediment cores were uncoupled from the recirculation system, closed airtight for a period long enough to measure steady changes in oxygen and nutrient concentrations while ensuring that oxygen concentration in the sediment-overlying sea water did not drop below 50% saturation. Oxygen concentration in the sediment-overlying sea water was continuously monitored with Oxygen Spot Sensors (OXSP5, Pyroscience) glued to the inner wall of the tubes. The sensors were operated with an optical oxygen meter (FireStingO2, Pyroscience), connected with a lens-spot adapter (SPADLNS) and Spot Fiber (SPFIB). Incubation time depended on the sediment type and temperature (i.e. longer incubation times for permeable sediment and measurements at low temperatures). Tubes of half the volume of a normal fine sandy sediment tube (i.e. 12.5 cm height) were incubated with tank water to estimate the influence of processes occurring only in the water column. Six (February) or five (April) times, bottom water samples were taken with a glass syringe for the determination of oxygen concentrations (in 12 mL Winkler bottles) and DIN (10 mL), the latter filtered through Whatman GF/F filters. At the same time, tank water was carefully injected into the overlying water of the tubes to compensate for the sampled volume. Tank samples were taken for oxygen and DIN analyses to correct for the dilution in the tubes during this additional sampling. Oxygen samples were stored in the same temperature-controlled room in the dark until further analysis (within 3 days); DIN samples were stored frozen (−20°C).

At the end of the incubations, the tubes were opened and the sediment was subsampled for pigments and organic carbon and nitrogen (each 2 ml from upper 2 cm). The remaining sediment was sieved on a 1 mm mesh to sample the macrofauna, which was preserved in ethanol. Macrobenthos specimens were identified to the lowest possible taxonomic level (typically species level), counted and biomasses were determined (blotted wet weights). Apart from deriving the structural characteristics, density, biomass and species richness, also the functional descriptors Bioturbation Potential index (BPi) and Bioturbation Potential of the Community (BPc) were calculated [37], [38].

### Laboratory analyses and flux calculation

Pigments were determined by HPLC (Gilson) analysis according to Wright and Jeffrey [39]. Total organic C and N content was analyzed with an Element Analyzer N1500 (Carlo Erba). Oxygen was analyzed by automated Winkler titration [40] and DIN by automated colorimetric techniques (SKALAR). Oxygen and DIN fluxes were calculated from the significant regression slopes of concentration over time compensating for dilution by refill water. Finally, a correction for the processes occurring in the water column was made, by subtracting the rates measured in the water incubations from the total measured rate.

### Mass budget modeling

The fluxes of O_2_, NO_x_, and NH_x_ across the sediment–water interface were used to estimate rates of nitrification, denitrification and total nitrogen mineralization. This was done by constructing an integrated mass balance of oxygen, nitrate and ammonium over the entire sediment column [29]. See Materials S1 for detailed methodology.

It must be noted that the DIN concentrations of the ‘tank water’ used in the core incubations (cf. Supra: 2.3 Sediment core incubations) differed from the field DIN concentrations [41]: while NH_x_ concentrations were rather similar in the field and in the lab, NO_x_ concentrations were one order of magnitude higher in the tank than in the field. The reported NO_x_ fluxes should therefore be considered as “potential fluxes”.

We chose to make measurements of several aspects of the N cycle rather than analyze a large number of replicates of fewer variables using a variance based statistical approach. The latter strategy allows for detection of significant differences in the measured variables, but we aimed to obtain a more holistic view by performing measurements of different N-cycle related processes covarying with ammonium fluxes (nitrate fluxes, oxygen fluxes) in combination with modeling of individual flux terms subjected to overall mass balance constraints. This allowed us to (1) assess the robustness of single measurements and (2) understand why patterns were observed.

### Statistical approach

To test differences in oxygen and pH profiles, a multivariate data matrix was constructed in which each depth horizon is considered a “variable” and each pH measurement a measure of “abundance” [24]. As not all pH profiles were measured to the same depth, including all data would make the design too imbalanced. Therefore, only the major depth horizons were considered (sediment–water interface till 5 mm below the sediment–water interface, in steps of 1 mm). Permutational ANOVAs (Permanova) were carried out to test for differences in these sediment oxygen and pH profiles. A Euclidean distance similarity matrix was built and subsequently fully crossed 3-way Permanova's were run with factors Month, Sediment and pH Treatment. To test the effect of these factors on macrobenthic community structure, a Bray-Curtis similarity matrix was constructed. In case of significant interaction of factors, pair-wise tests were run to further investigate the observed differences. For sediment oxygen and pH profiles, SIMPER analysis was used to determine which depth was responsible for any differences identified, whereas for macrobenthic community analysis, this test indicated the species that characterized the community. Homogeneity of multivariate dispersion (‘variance’) was tested with PERMDISP for any of the significant terms in Permanova; if significant, this test indicates that observed patterns can be a result of both treatment and dispersion. The difference in pH in the water tanks during the acclimatization period was also tested with Permanova, using a two-factor design with pH treatment and time.

While testing the effect of pH Treatment, Sediment and Month on the univariate variables chlorophyll-*a*, SCOC, DIN fluxes, estimated nitrification, total N mineralization and macrobenthic characteristics, PERMDISP often pointed at heterogeneity of variances that complicated the interpretation of the significant Permanova's. Therefore, we adopted a linear model with a generalized least-squares extension [42]–[44], which allows unequal variances among treatment combinations to be modeled as a variance covariance matrix [42], [43]. Following West et al. [42] and Zuur et al. [44], the most appropriate variance covariate matrix was determined using AIC scores in conjunction with plots of fitted values versus residuals with different variance covariate terms relating to the independent variables, using restricted maximum likelihood (REML, [42]). This procedure resulted in the use of a variance structure that allowed for different variances per stratum for sediment type and pH treatment (varIdent function, R package nlme). The fixed component of the model was then refined by manual backwards stepwise selection using maximum likelihood (ML) to remove insignificant variable terms. Following Underwood [45], the highest order significant interactions in the minimal adequate model were examined, but the nested levels within these were not. The importance of the highest order term was estimated using a likelihood ratio (L-ratio) test to compare the full minimal adequate model with a model in which the relevant variable and all the interaction terms that it was involved in, was omitted. Pair-wise tests were carried out within the R package contrast [46].

All multivariate analyses were carried out within PRIMER v6.0 with Permanova+ add-on software [47], [48]. Univariate models were performed in the free statistical environment R (http://cran.r-project.org). Results are shown as mean ± standard deviation.

## Results

### Evaluation of experimental system

A significant difference in pH of 0.31±0.03 between control and acidified treatments was established in the water tanks in both months (Permanova, pseudo-F >36.8, p = 0.001) (Table S1). Temperature and salinity remained constant and oxygen concentration stayed saturated over time. All other seawater carbonate variables remained constant over time as well (Table S1).

### Pigments

Chlorophyll-*a* concentrations were affected by the interactive effects of pH Treatment, Sediment and Month (Table 1, 2). In the fine sandy sediments, chl-*a* concentrations were low in February. The bloom deposition was evidenced by higher chl-*a* concentrations at the sediment in April. Chlorophyll-*a* concentrations in permeable sediments were an order of magnitude lower in February, but also increased in April (Table 1, 2).

**Table 1:** Results of Generalized Least Squares for differences in chlorophyll-a content of the sediment, macrobenthic univariate descriptors and measured and estimated sediment processes among the experimental factors pH treatment, Sediment and Month.

	Factor	L-ratio	p-value
Chlorophyll-*a*	pH×Sediment×Month	7.01	0.008
Macrobenthic density	Sediment	13.12	<0.001
	Month	4.63	0.03
Macrobenthic species richness	Sediment	10.28	0.001
Macrobenthic biomass	Sediment	10.33	0.001
Bioturbation potential, BPc	Sediment	12.06	<0.001
	Month	4.09	0.043
SCOC	pH	5.44	0.02
	Sediment×Month	30.48	<0.001
NH_x_ effluxes	Month	6.42	0.011
	Sediment	29.37	<0.001
NO_x_ effluxes	Sediment×Month	9.48	0.002
Nitrification	pH×Month	8.07	0.004
	Sediment×Month	18.68	<0.001
Total N mineralization	Sediment×Month	17.10	<0.0001

Only significant results (p<0.05) are shown.

**Table 2:** Average ± sd of sediment chlorophyll-*a*, measured and estimated sediment processes and macrobenthic parameters for each of the factors (pH treatment, Month, Sediment or their interactions) with a significant effect.

	pH		Month	Month×pH		Sediment	Sediment×Month		Sediment×Month×pH			
**Chlorophyll-** ***a***	**-**		**-**			**-**			**Permeable**	February	Control	0.05±0.07
											Acidified	0.02±0.03
										April	Control	0.13±0.07
											Acidified	0.13±0.04
									**Fine sandy**	February	Control	0.29±0.04
											Acidified	0.68±0.33
										April	Control	0.81±0.47
											Acidified	11.03±4.48
**SCOC (mmol O_2_ m^−2^ d^−1^)**	**Control**	11.73±8.09	**-**			**Permeable**	February	5.87±4.98	**-**			
	**Acidified**	6.63±7.45					April	1.25±2.36				
						**Fine sandy**	February	10.33±3.57				
							April	19.26±3.21				
**NHx efflux (mmol N m^−2^ d^−1^)**	**-**		**February**		1.09±1.55	**Permeable**		0.31±0.39	**-**			
			**April**		1.00±1.00	**Fine sandy**		1.79±1.41				
**NOx efflux (mmol N m^−2^ d^−1^)**	**-**		**-**			**Permeable**	February	8.66±1.22	**-**			
							April	0.25±0.47				
						**Fine sandy**		3.04±2.53				
**Nitrification (mmol N m^−2^ d^−1^)**	**-**		**February**	Control	3.74±2.21	**Permeable**	February	3.15±2.36	**-**			
				Acidified	0.22±1.14		April	0.02±0.87				
			**April**		0.87±1.24	**Fine sandy**		1.17±1.72				
**N mineralization (mmol N m^−2^ d^−1^)**	**-**		**-**			**Permeable**	February	4.88±1.23	**-**			
							April	0.42±0.64				
						**Fine sandy**		2.97±0.84				
**Density (ind. m^−2^)**	**-**		**February**		725±879	**Permeable**		44±41	**-**			
			**April**		370±412	**Fine sandy**		1050±659				
**Biomass (g m^−2^)**	**-**		**-**			**Permeable**		3±5	**-**			
						**Fine sandy**		189±137				
Species richness (core^−1^)*	**-**		**-**			**Permeable**	*per 283.53 cm^2^	1.13±0.99	**-**			
						**Fine sandy**	*per 78.54 cm^2^	3.13±1.25				
**BPc (m^−2^)**	**-**		**February**		898±1102	**Permeable**		57±51	**-**			
			**April**		601±671	**Fine sandy**		1442±785				

### Macrobenthos

Macrobenthic community structure was only dependent on sediment type (Permanova, pseudo-F = 9.29 p = 0.001) and the two sediment types were strongly dissimilar in terms of macrobenthic community structure (99.91%; SIMPER analysis). The fine sandy sediment community was dominated (94% in terms of numbers) by the small surface-modifying *Magelona johnstoni* and the biodiffusing *Scoloplos armiger* polychaete species and the surface-modifying bivalve *Macoma balthica* (SIMPER analysis; for functional group classification see [38]). The permeable sediment community consisted 94% of the biodiffusing polychaetes *Nephtys cirrosa* and *Ophelia limacina* and the surface-modifying amphipod *Urothoe brevicornis* (SIMPER analysis). Neither structural (density, biomass, species richness) nor functional (BPc) univariate macrobenthic characteristics were affected by pH treatment. Density, biomass, species richness and BPc only differed among sediments (Table 1). The fine sandy sediment clearly displayed higher macrobenthic density, species richness, biomass and BPc values than the permeable sediment (Table 2). Density and BPc differed also marginally among months (Table 1): in February, density and BPc were higher than in April (Table 2).

### pH and oxygen sediment profiles

**Figure 2:**
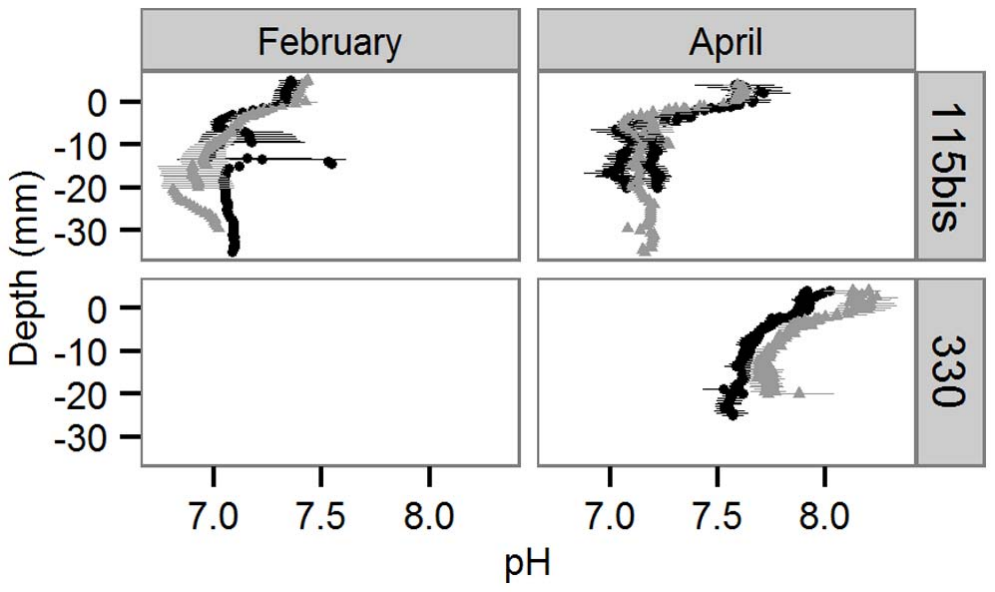
Sediment pH profiles. Mean ± sd pH in control (▴) and acidified (•) treatments of the two sediment types (115bis: fine sandy; 330: permeable) and two seasons (February and April). No data for permeable sediment in February.

pH profiles in the upper 5 mm of the sediment (Fig. 2) were significantly affected by the interactive effects of Sediment and pH (Permanova: pseudo-F = 17.31, p = 0.001) and Month and pH (Permanova: pseudo-F = 4.23, p = 0.001). However, for both tests, some heterogeneity of variances was detected (Permdisp: p = 0.001), which indicates that there is also a significant dispersion effect. Pair-wise tests revealed significant differences between pH profiles of acidified and control treatments in permeable sediments (p<0.05), and a marginally significant difference (p = 0.054) between pH profiles of acidified and control treatments in April. The surface and the first two millimeters made up 60% of the difference between pH profiles of acidified and control permeable sediments (Simper). In these layers, the pH was on average 0.24±0.03 units lower than in the respective control surface sediment layers. In April, the difference between acidified and control sediments was mainly (54%) found in the deeper 3–5 mm, where pH values were 0.03±0.03 units lower in the acidified than in the control sediment.

**Figure 3:**
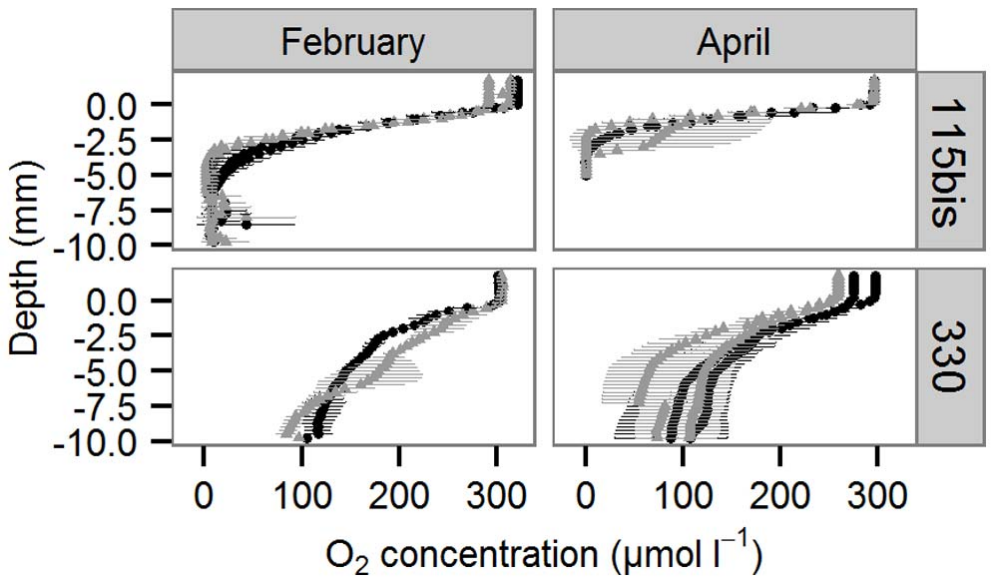
Sediment O_2_ profiles. Mean ± sd O_2_ concentration in control (▴) and acidified (•) treatments of the two sediment types (115bis: fine sandy; 330: permeable) and two seasons (February and April).

Figure 3 suggests a deeper sediment oxygen penetration in acidified fine sediments in February than their respective controls, but these effects were not significant. Only the interactive effects of Month and Sediment influenced sediment oxygen penetration marginally (Permanova: pseudo-F = 2.90, p = 0.042), where the oxygen content of fine sandy sediments was higher in February than in April (pair-wise tests: p<0.05) and that of permeable sediments stayed equal (pair-wise tests: p>0.05).

### Fluxes at the sediment-water interface

**Figure 4:**
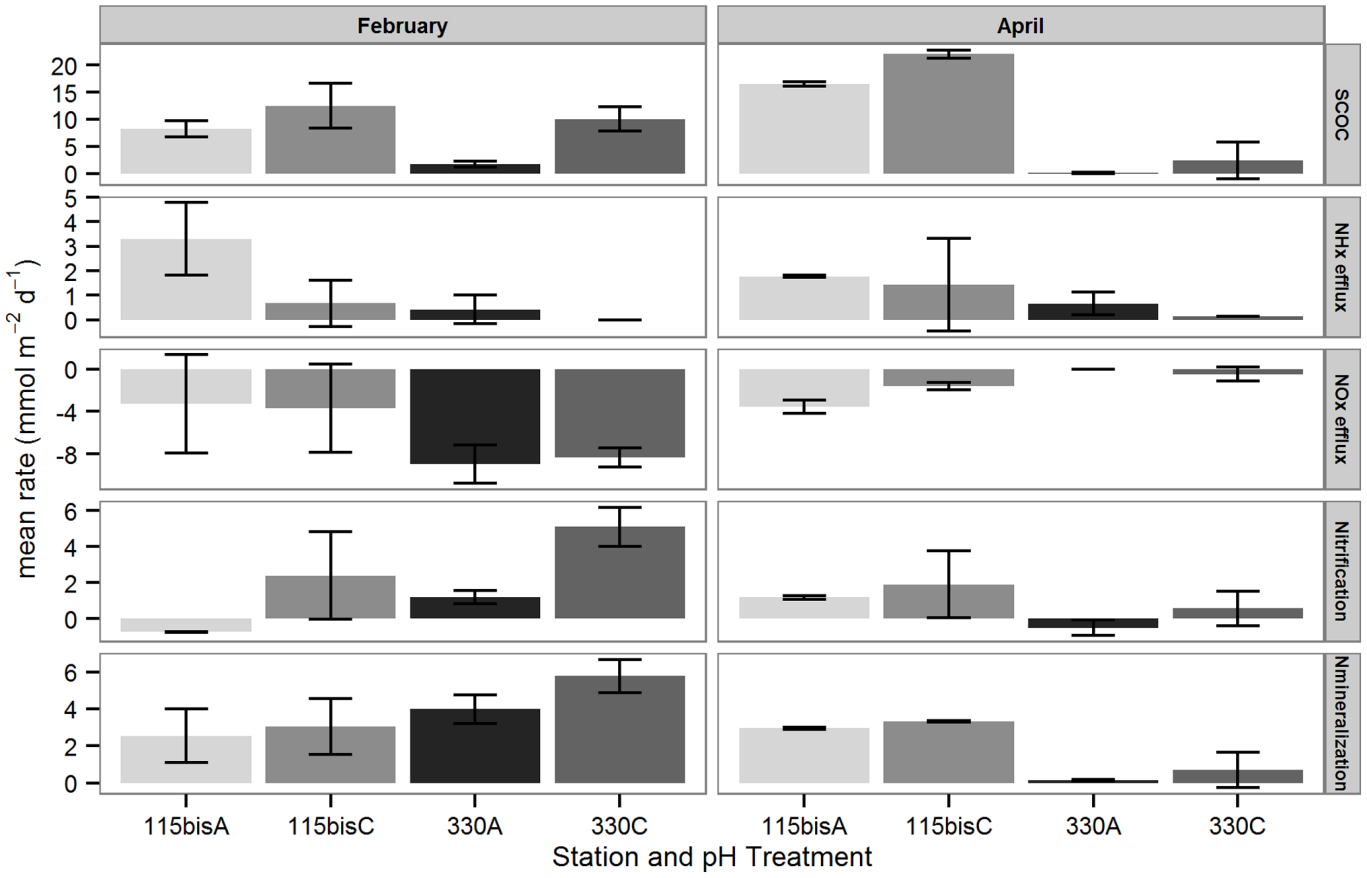
Sediment–water fluxes. Measured Sediment Community Oxygen Consumption (SCOC, mmol O_2_ m^−2^ d^−1^), NH_x_ and NO_x_ effluxes and estimated nitrification and total N mineralization (mmol N m^−2^ d^−1^) (mean ± sd) from control and acidified treatments of the two sediment types (115bis: fine sandy; 330: permeable) and two seasons (February and April).

SCOC differed among pH treatment (Table 1, 2; Fig. 4), and was significantly lower in acidified treatments (59±35%) than in the respective controls. SCOC also differed among sediments and months (Table 1, 2). Significantly higher SCOC was measured in February compared to April in the permeable sediments (pair-wise tests: p<0.002). In the fine sandy sediments, SCOC was higher in April than in February (pair-wise tests: p<0.02).

All NH_x_ fluxes were directed towards the water column, and only differed between sediments (Table 1, 2; Fig. 4), with lower effluxes in permeable than in fine sandy sediment, and between months (Table 1, 2), with slightly higher effluxes in February than in April. Also note the high variability among NH_x_ effluxes, especially in the acidified treatments (Fig. 4).

NO_x_ fluxes were mainly directed into the sediment and differed only among months and sediments (Table 1, 2; Fig. 4). In the permeable sediments, NO_x_ influxes were highest in February compared to April (pair-wise tests: p<0.05), whereas they were equal in both months in the fine sandy sediments (pair-wise tests: p>0.05).

The nitrification rates estimated by the mass budgets were affected by the interactive effects of pH Treatment and Month and Sediment and Month (Table 1, 2; Fig. 4). Only in February, estimated nitrification rates were significantly reduced by 94% in sediments underlying an acidified water column compared to control sediments. Nitrification estimates were significantly higher in permeable sediments in February compared to April.

Total N mineralization estimates were only affected by the interactive effects of Sediment and Month (Table 1, 2; Fig. 4). Total N mineralization estimates were significantly higher in permeable sediments in February compared to April.

## Discussion

Our results indicate that ocean acidification in coastal sediments reduces sediment community oxygen consumption and nitrification, but does not significantly affect total N mineralization. To the best of our knowledge, this is the first observation of reduced sediment community oxygen consumption (60% reduction) in sediments underlying an acidified water column. SCOC is the sum of all oxygen consuming processes in the sediment, which include nitrification, oxidation of reduced substances other than ammonia and nitrite, but also the oxygen uptake of the fauna present and bacteria, whether or not stimulated by macrofaunal bio-irrigation. The effect of ocean acidification on faunal stimulation of SCOC through bio-irrigation is expected to be minor, since no real bio-irrigators were present in the macrofauna communities and such effects are only expected on the longer term (at least several weeks) [19], [21], [22]. As total N mineralization remained constant, the decrease in SCOC can be mainly attributed to a lower demand by nitrifying organisms. This effect was found in two biologically and biogeochemically contrasting sediments that were incubated at a realistic scenario of reduced seawater pH by 2100. A 94% reduction in nitrification rate was observed before but not during the annual phytoplankton bloom deposition.

Our results are in contrast with the two earlier studies that did not find a significant reduction in ammonia oxidation (which is the first step in nitrification) in the North Sea, in muddy sediments in January (pre-bloom) [17], nor in non-bioturbated muddy surface sediments in September (post-bloom) [18], nor in permeable sediments in August (post-bloom) [17], [18]. These different seasonal and sediment settings might complicate the comparison with our study. On the other hand, other North Sea studies interpreted fluxes of reduced and oxidized N-species across the sediment–water interface, and suggested a decreased benthic nitrification mainly at much more extreme pH reductions (pH range: 5.6–7.6) [19], [20], [23], [24] than we applied.

The potential to detect significant trends at realistic acidification scenarios depends on the accuracy of the applied methods. Measuring fluxes in closed tube incubations and correcting for processes occurring in the water column offers the advantage of a holistic approach and balanced interpretation of the on-going processes in each sediment core, but there still exists large variability between replicate experimental units (see relatively large error bars on our figures – but nonetheless are the fluxes in this study in the same range as fluxes earlier measured in the area [41] - and the range of variation in Kitidis et al. [17]). As such, the lower nitrification rates in April, especially in permeable sediments, combined with a relatively high variability among replicates, probably prevent a statistically detectable decrease in April. In some acidified treatments (fine sandy sediment in February, permeable sediments in April), estimated nitrification rates were slightly negative, indicating a violation of the mass budget assumption that all reduced substances other than ammonia are reoxidized within the sediment [41]. This suggests that, in addition to ammonia and nitrite oxidation, other oxidation processes are affected by acidification as well. This could have included NH_4_^+^ efflux rates, which were also highly variable among replicates. More dedicated experiments, where also the fluxes of other reduced substances are measured and replication is increased, are needed to enhance our understanding of stressor effects (e.g. acidification) on benthic ecosystem functioning.

How can the observed decrease in estimated nitrification rates be explained? The lower pH imposed on the sea water reduced nitrification in both our sediments, by altering the pH within the sediment matrix. In the fine sandy sediments, we observed steep pH profiles in accordance with earlier results [20], [24], [49]. These profiles are driven by microbially mediated redox reactions linked to mineralization of organic matter and the dissolution and precipitation of minerals such as CaCO_3_ (reviewed in [10]). Hence, benthic microbial communities involved in nitrification in fine sandy sediments are likely more adapted to low pH. In addition, the dissolution of minerals acts as a buffer towards pH changes in the water column [10], [50]–[52]. The very similar pH profiles in our fine sandy acidified and control sediments suggest that these sediments were buffered against water column acidification, which resulted in relatively small effects. In contrast, the pH in the permeable sediment was consistently lowered over the upper 1 cm of the sediment under the acidification scenario (Fig. 3). These pH changes may affect biogeochemical rates in several ways. First of all, the first step in nitrification (ammonia oxidation) requires NH_3_ rather than NH_4_^+^ [53], [54]. Since seawater buffers pH by shifting the NH_3_/NH_4_^+^ balance towards the larger NH_4_^+^ ion at lower pH, a decrease in substrate for ammonia oxidation takes place during ocean acidification. In the presented experiments, a decrease in pH of 0.3 would imply a substrate decline of about 50% [55]. Secondly, large infaunal bioturbators can stimulate nitrification [56], but when these are negatively affected by pH reductions in the overlying water, the stimulating effect disappears and a negative effect on ammonia oxidising microbial organisms is observed [18]. Bioturbation is an instrumental mechanism for ammonia-oxidizing bacteria (AOB) but not for ammonia-oxidizing archaea (AOA) [57]. Consequently, bioturbation-mediated change in AOB communities has been suggested to explain the outcome of ocean acidification effects on ammonium oxidation in a pH range of 7.90–6.80 [18]. Quantification of faunal activity (bioturbation and bio-irrigation) was not specifically targeted in our experiments but we did not observe effects of acidification on the structural and functional characteristics of the macrofauna communities in our sediments, which were not inhabited by strong bio-irrigators or bioturbators. Moreover, bioturbation is not expected to decrease in short-term experiments at realistic pH declines [19], [21], [22].

We speculate that the observed reduction in nitrification in the present short-term study could result from rapid acidification-induced changes in microbial activity and composition, particularly in terms of the realized ratio of archaea to bacteria [58], [59]. Within buffered cohesive sediments, only minor changes in the active benthic total microbial communities from surface sediments were observed in a 2-week acidification experiment [60]. However, in the same experiment, Tait et al. [61] revealed changes in the relative abundance of benthic AOB and AOA under reduced pH circumstances. In addition, under strong acidification scenarios, sedimentary AOB show drastic reductions in amoA transcription, while sedimentary AOA transcripts increase [61]. This suggests that AOB and AOA in marine sediments have different pH optima, and the impact of elevated CO_2_ on N cycling may be dependent on the relative abundances of these two major microbial groups [61].

Finally, we should be aware that short-term effects of acidification do not necessarily reflect the net effect of climate forcing; long-term studies more likely include seasonal and other natural variability that bioturbators and the interaction that species undergo [22], [62]. As benthic nitrification makes up most of ocean nitrification, a reduction of this nitrogen cycling in both permeable and fine sediments in winter, when coastal benthic nitrification is generally highest [63], [64], could have global-wide impacts on coupled nitrification-denitrification and hence eventually on pelagic nutrient availability. High NH_x_ effluxes could as such precipitate a positive feedback on acidification, if eutrophication is accelerated by the NH_x_ flux, via oxidation of senescent phytoplankton biomass [65].

## Supporting Information

Table S1 **Monitoring data of 14 days prior to the incubations.** Minimum, maximum, average and standard error of the monitored variables and calculated variables (pCO2, DIC and Ωaragonite and Ωcalcite) in the tanks of the different experiments.
(DOCX)

Materials S1 **Mass Budget Modelling.**
(DOCX)
