# Supplementary material for: Empirical Evidence Reveals Seasonally Dependent Reduction in Nitrification in Coastal Sediments Subjected to Near Future Ocean Acidification
Source: PLoS One. 2014 Oct 16;9(10):e108153. doi: 10.1371/journal.pone.0108153 (PMC4199590; doi:10.1371/journal.pone.0108153)
Supplement: Table S1 — Monitoring data of 14 days prior to the incubations. Minimum, maximum, average and standard error of the monitored variables and calculated variables (pCO2, DIC and Ωaragonite and Ωcalcite) in the tanks of the different experiments. (DOCX) [file pone.0108153.s001.docx]

Table S1: Monitoring data of 14 days prior to the incubations.

Minimum, maximum, average and standard error of the monitored variables and calculated variables (pCO_2_, DIC and Ω_aragonite_ and Ω_calcite_) in the tanks of the different experiments.

| Month |  | February | February | April | April |
| --- | --- | --- | --- | --- | --- |
| pHTR |  | A | C | A | C |
| pH | min | 7.59 | 7.84 | 7.67 | 8.04 |
|  | max | 8.01 | 8.07 | 7.76 | 8.11 |
|  | average | 7.68 | 7.99 | 7.73 | 8.07 |
|  | se | 0.11 | 0.07 | 0.03 | 0.03 |
| Temperature (°C) | min | 5.9 | 5.8 | 9.5 | 9.3 |
|  | max | 6.3 | 6.3 | 9.7 | 9.7 |
|  | average | 6.1 | 6.0 | 9.6 | 9.5 |
|  | se | 0.1 | 0.1 | 0.1 | 0.1 |
| Salinity | min | 31.70 | 31.90 | 30.60 | 30.90 |
|  | max | 34.00 | 34.00 | 31.10 | 31.90 |
|  | average | 32.33 | 32.65 | 30.87 | 31.39 |
|  | se | 0.78 | 0.67 | 0.18 | 0.35 |
| [O_2_] (µmol l^-1^) | min | 280.60 | 296.60 | 264.90 | 288.90 |
|  | max | 320.80 | 317.60 | 290.10 | 301.30 |
|  | average | 293.65 | 312.28 | 274.03 | 293.50 |
|  | se | 13.81 | 6.72 | 8.22 | 3.95 |
| A_T_ (µmol kg^-1^) | min | 2822.01 | 2799.93 | 2703.38 | 2646.09 |
|  | max | 3034.67 | 2972.55 | 2807.72 | 2749.82 |
|  | average | 2909.52 | 2849.30 | 2744.16 | 2696.80 |
|  | se | 66.90 | 58.24 | 36.54 | 43.20 |
| pCO_2_ (µatm) | min | 533.57 | 458.93 | 1027.50 | 400.46 |
|  | max | 1618.92 | 841.44 | 1297.67 | 475.56 |
|  | average | 1381.38 | 571.91 | 1132.81 | 439.61 |
|  | se | 327.51 | 122.23 | 104.18 | 28.49 |
| DIC (µmol kg^-1^) | min | 2501.98 | 2478.31 | 2552.91 | 2322.07 |
|  | max | 2878.29 | 2679.95 | 2650.96 | 2409.27 |
|  | average | 2770.55 | 2542.70 | 2601.18 | 2367.06 |
|  | se | 113.29 | 67.19 | 38.05 | 30.12 |
| Ω_aragonite_ | min | 1.73 | 2.85 | 1.86 | 3.75 |
|  | max | 3.95 | 4.34 | 2.28 | 4.46 |
|  | average | 2.15 | 3.86 | 2.12 | 4.08 |
|  | se | 0.66 | 0.47 | 0.15 | 0.25 |
| Ω_calcite_ | min | 2.64 | 4.33 | 2.85 | 5.75 |
|  | max | 6.01 | 6.62 | 3.49 | 6.82 |
|  | average | 3.28 | 5.88 | 3.25 | 6.24 |
|  | se | 1.00 | 0.72 | 0.23 | 0.37 |
