# Supplementary material for: Empirical Evidence Reveals Seasonally Dependent Reduction in Nitrification in Coastal Sediments Subjected to Near Future Ocean Acidification
Source: PLoS One. 2014 Oct 16;9(10):e108153. doi: 10.1371/journal.pone.0108153 (PMC4199590; doi:10.1371/journal.pone.0108153)
Supplement: Materials S1 — Mass Budget Modelling. (DOCX) [file pone.0108153.s002.docx]

Materials S1. Mass budget modelling

Oxygen is either directly consumed to oxidize organic carbon (oxic mineralization - OxicMin), or indirectly through the re-oxidation of reduced substances formed by anoxic mineralization (AnoxicMin). Part of the reduced substances remain buried in the sediment (pSolidDepo) and are not re-oxidized. We assume that one mole of oxygen is consumed for each mole of carbon originally mineralized (respiratory quotient = 1). Ammonium results from the mineralization of organic nitrogen (Nmineralization), whilst it is consumed by nitrification, which requires two moles of oxygen for each mole of ammonium. Denitrification consumes 0.8 moles of NO_3_ for one mole of carbon denitrified. Oxygen, nitrate, and ammonium are further exchanged through the sediment–water interface (O_2_influx, NO_x_influx, NH_x_influx), while the lower boundary of the sediment is assumed to be a no flux boundary.

The resulting balances are summarized below.

$$\frac{\mathrm{dO}_{2}}{\mathrm{dt}}= O_{2}influx-OxicMin-AnoxicMin*\left( 1-pSolidDepo \right)-Nitrification*2$$

$$\frac{\mathrm{dNH}_{x}}{\mathrm{dt}}= \mathrm{NH}_{x}influx+Nmineralization-Nitrification$$

$$\frac{\mathrm{dNO}_{x}}{\mathrm{dt}}= \mathrm{NO}_{x}influx+Nitrification-Denitrification*0.8$$

The fluxes across the sediment–water interface (O_2_inFlux, NH_x_inFlux, NO_x_inFlux) were estimated during the incubation experiments, while the rate of change of oxygen, nitrate and ammonium fluxes was assumed to be zero (geochemical steady state reached after two weeks equilibration). With six remaining unknowns (OxicMin, AnoxicMin, Nmineralization, Nitrification, Denitrification, pSolidDepo) and only three equations, the mass balance model is not closed. We therefore make the assumption that the burial of anoxic substances can be ignored (pSolidDepo = 0). This allows combining the oxic and anoxic mineralization into one quantity (OxicAnoxicMin). The extra equation to balance the model then imposes a relationship between nitrogen and carbon mineralization, using the average N:C ratio as measured in the surface sediment of each station and each month. The mass balances then become:

$$0= O_{2}influx-OxicAnoxicMin-Nitrification*2$$

$$0= \mathrm{NH}_{x} influx+Nmineralization-Nitrification$$

$$0= \mathrm{NO}_{x}influx+Nitrification-Denitrification*0.8$$

Where:

$Nmineralization=\left( OxicAnoxicMin+Denitrification \right)*N:Cratio$

With three equations and three unknowns this makes the model evenly determined. These three equations can be solved for the three unmeasured quantities (OxicAnoxicMin, Nitrification, Denitrification). The mass balance modeling was performed using package limSolve [1] available in the open source software R (R development team, 2008).

**References**

[1] Soetaert K, Van den Meersche K, van Oevelen D (2009) limSolve: Solving linear inverse models. R Package Version 1. Available: http://cran.r-project.org/web/packages/limSolve/.
